# Joint examination of reflexive vertical saccades and small involuntary fixational saccades improves the classification of patients with progressive supranuclear palsy (PSP): a ROC study

**DOI:** 10.1007/s00221-025-07031-w

**Published:** 2025-03-13

**Authors:** Wolfgang Becker, Olga Vintonyak, Jan Kassubek

**Affiliations:** 1 Section of Neurophysiology, Department of Neurology, University of Ulm, Oberer Eselsberg 45, 89081 Ulm, Germany; 2 Department of Neurology, University of Ulm, Ulm, Germany

**Keywords:** Video-oculography, Small involuntary fixation saccades, Visually guided reactive saccades, Progressive supranuclear palsy (PSP), Receiver operator characteristic, Discrimination between PSP and healthy subjects

## Abstract

A slowing of saccadic eye movements is one of the key symptoms of progressive supranuclear palsy and therefore represents a core functional domain of the current diagnostic criteria. However, there is considerable overlap between the saccade velocities of healthy people and patients in early stages. Therefore, a highly specific discrimination between patients and controls based on eye velocity often results in a considerable loss of sensitivity. Another symptom of progressive supranuclear palsy is a high frequency of square wave jerks formed by small involuntary fixational saccades. Using ROC analyses of 50 patients and 50 controls and focusing on points of 100 and 90% specificity or sensitivity, we investigated whether the velocity and gain data of visually guided reflexive saccades could be combined with each other and with parameters of fixational saccades to improve discriminability compared to considering saccade velocity alone. Both approaches were successful in patients with long disease duration but less so in cases of short duration. The displacement rate produced by square waves during fixation proved helpful because its frequency distributions in patients and controls had value ranges that were not shared by the two groups. This fact allowed an a priori classification of some subjects as either patients or controls. Modified ROC analyses using this a priori information are expected to work equally well in patients with short and long disease duration. In future studies it might be addressed if these methods can also improve the discrimination between PSP and other Parkinsonian disorders.

## Introduction

Progressive supranuclear palsy (PSP) is a neurodegenerative disorder named after one of its prominent clinical characteristics, an impairment primarily of vertical eye movements (Steele et al. 1964). PSP is a 4R-tauopathy predominated by subcortical pathology in neurons, astrocytes and oligodendroglia, which is associated with various clinical phenotypes (Kovacs et al. 2020). To capture the multifaceted phenotypical presentations of PSP, four functional domains (ocular motor dysfunction, postural instability, akinesia, and cognitive dysfunction) have been defined in the current Movement Disorder Society (MDS) diagnostic criteria for PSP (Höglinger et al. 2017). Within each of these domains, three clinical features have been proposed that contribute different levels of diagnostic certainty (probable, possible, suggestive). In the oculomotor domain, these are (1) vertical supranuclear gaze palsy, (2) slow velocity of vertical saccades and (3) enlarged fixational saccades with frequent square wave jerks (SWJ). Symptoms (1) and (2) result from supranuclear degeneration which to a lesser extent also affects the patients’ horizontal eye movements (Bhidayasiri et al. 2001; Pinkhardt et al. 2008; Rivaud-Péchoux et al. 2000; Kumar and Chung 2014). The origin of symptom (3), the increased frequency and the enlargement of the mostly horizontal small involuntary fixational saccades (SIFS) away from target and back to target, is less well known and may be more diffuse. Frontal dysfunction could lead to disinhibition of the superior colliculus via the substantia nigra pars reticulata (Otero-Millan et al. 2011), thereby increasing the frequency of SIFS, and a loss of vertical inhibitory burst neurones has been implicated in the enlargement of SIFS (Otero-Millan et al. 2013). SIFS are increased by a factor of two or more in PSP compared to healthy people and can reach amplitudes of up to 3° (Becker et al. 2023; Pinnock et al. 2010; Troost and Daroff 1977). When subsequent SIFS have similar amplitudes and motion planes, they form SWJ. As the likelihood of SWJ increases with SIFS amplitude, SWJ are more common in PSP patients than in healthy people (Alexander et al. 2018; Becker et al. 2023; Chen et al. 2010; Otero-Millan et al. 2011, 2013; Rascol et al. 1991). Thus, in addition to the enlargement of SIFS, a frequent occurrence of SWJ is also characteristic of PSP. Note that SIFS are often referred to as saccadic intrusions, a term we avoid because of its connotation of disruption (Becker et al. 2023).

In clinical settings, the presence of the eponymous vertical palsy is mostly noted upon bedside examination and may be overlooked if it is an only mild one, particularly if patients lack the fractionation of saccadic responses to target steps observed in more severe cases (Gorges et al. 2014). It is even more difficult on the bedside to assess whether a patient's fixational saccades are enlarged. Therefore, quantitative methods, mostly based on video-oculography (VOG), have come increasingly into use as diagnostic tools (Chen et al. 2010; Habibi et al. 2022; Herwig et al. 2021; Marx et al. 2012; Pinkhardt et al. 2008; Wunderlich et al. 2021). Distinguishing patients from healthy subjects on the basis of oculomotor results is a binary detection problem. Optimal solutions for such problems can be searched with the help of ROC curves reflecting the receiver-operator characteristics of the respective problem (Habibzadeh et al. 2016). These curves describe the opposite variation of sensitivity and specificity as a function of the cut-off value chosen for the parameter that is supposed to discriminate the two groups. In the context of PSP detection, ROC analyses have recently been invoked by Wunderlich et al. (2021) and Quattrone et al. (2022). However, these authors only considered ROC curves after choosing cut-off values based on a predefined distance of the discrimination parameter from its central value in controls (e.g. *z* = − 2.5). In contrast, like Herwig et al. (2021), Marx et al. (2012) and Otero-Millan et al. (2011), we here consider ROC curves as a means of searching for cut-off values that satisfy predefined goals, such as maximising either specificity or sensitivity or for searching a statistically optimal trade-off between specificity and sensitivity. We apply this method to a variety of possible discrimination parameters such as the velocity and gain of reactive saccades and the amplitude and displacement rate of fixational saccades. Moreover, we explore the possibility that different discrimination parameters can be combined to improve discriminability. In particular, we were interested in (1) whether the parameters of SIFS could be used as an additional source of information that would help to sharpen the discrimination between patients and controls achievable with saccade velocity or gain alone and (2) how this would compare with the improvements from multiplicative combinations of velocity and gain as reported by Quattrone et al. (2022). A successful improvement in the differentiation between PSP patients and healthy people may also provide a perspective for the not always easy but clinically more important challenge of differentiating PSP from Parkinson’s disease (PD).

## Methods

### Participants

This report is a retrospective study based on data from 50 patients and 50 age-matched controls without known neurological affections (Table 1). The diagnosis of recent PSP cases was based on the MDS criteria for PSP (Höglinger et al. 2017) which were also retrospectively applied to the cases diagnosed originally according to the NINDS-SPSP^1^ criteria (Litvan et al. 1996). The oculomotor data of the PSP patients were obtained during a standardised oculomotor test battery which was administered routinely as part of the patients' general clinical work-up. Control subjects were recruited among patients’ relatives and acquaintances of the authors and were presented with the same test protocol as patients. The two cohorts partially overlap with those of a previous study (Becker et al. 2023). The study had been approved by the Ethics Committee of the University of Ulm (reference #76/20), and subjects had given their written consent in accordance with the Declaration of Helsinki.

**Table 1:** Demographic and clinical data

		CTR	PSP
	N	50	50
Age/yrs	Median	67	71
	Min–max	51–78	52–80
m/f ratio		26/24	24/26
UPDRS	Median	–	31
	Min–max	–	15–50
Disease duration	Median	–	3
	Min–max	–	1–8

*N* number of subjects

### Equipment, procedures and data processing

The recording equipment, experimental procedures and data processing methods have been described in detail previously (Becker et al. 2023; Gorges et al. 2014; Wunderlich et al. 2021). Briefly, participants faced a hemicylindrical screen at a distance of 1.5 m, which carried arrays of red LEDs subtending 0.3° that were lit according to the experimental protocol. First, participants were asked to fixate the central LED steadily for 32 s (recording of SIFS). They then tracked a pseudorandom sequence of horizontal steps of the target LED with amplitudes of 5, 10, 20 and 40°, each step starting from the target position reached by its predecessor, and then a similar sequence of vertical up and down steps of 5, 10, 15 and 30°; target eccentricity was limited to ± 20° horizontal and ± 15° vertical (recording of VGRS). The eye movements were recorded with a video-oculography system (EyeSeeCam^®^) which sampled the horizontal and vertical movements of both eyes at a frequency of 220 Hz and transferred them to MATLAB^®^ files.

Data analysis was performed using custom MATLAB^®^ scripts that orthogonalised the horizontal and vertical eye position records from both eyes, converted them to cyclopean signals, and displayed them on a computer screen. The SIFS recorded during the fixation task were identified by visual inspection based on their characteristic dynamic overshoots (cf. Fig.1 in Becker et al. 2023). Using a cursor, their onset and the end of their dynamic overshoot processes were marked by hairlines. The times and the horizontal and vertical eye positions read from these markers were stored in computer files for further processing. The first two seconds of the fixation period and epochs affected by artefacts were discarded. The VGRSs recorded during target step tracking were analysed by a visually supervised semi-automatic software that identified all saccades occurring after a target step (identification criteria: peak velocity > 20°/s, duration > 10 ms, amplitude > 0.5°). Usually, the first saccade with a latency of more than 200 ms after a target step was considered the primary saccade (for the problems associated with this definition see Limitations).

**Fig. 1:**
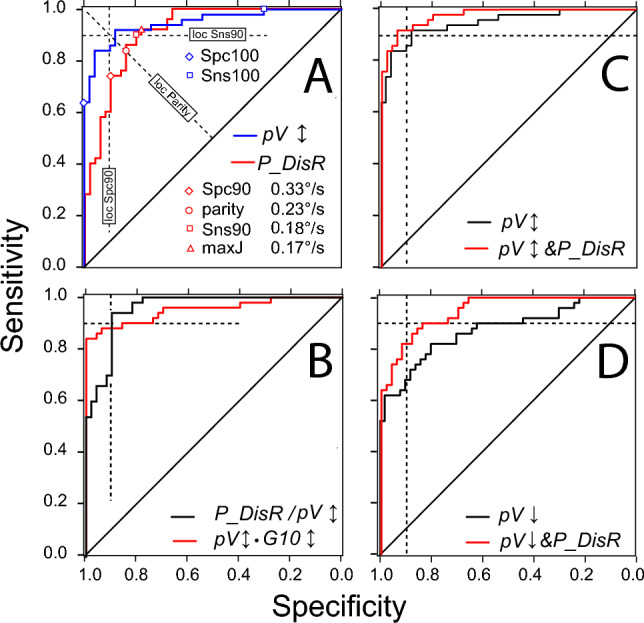
Receiver operator characteristic (ROC) curves. **A** comparison between the SIFS parameter *P*_*DisR* (red) and the VGRS parameter* pV↕* (blue). Dashed lines show the loci of the Spc90, parity and Sns 90 points. Red open symbols mark these points and the maxJ point on the ROC curve of *P_DisR*. The Spc100 and Sns100 points are always identical with the points where the ROC curves leave the left and the upper boundaries of the diagram, respectively (example marked by blue open symbols on the ROC curve of *pV↕*). Note that the values of parameter *P_DisR* decrease from their maximum at the lower left to their minimum at the upper right, whereas those of *pV↕* increase along this course. **B** comparison of the composite parameters *pV↕•G10↕* (red) and *P_DisR/pV↕* (black)*.* Note the very different behaviour of their ROC curves at the approach of Spc100 and Sns100 despite similar AuC (0.952 and 0.958), resulting in high Sns(Spc100) and low Spc(Sns100) values in *pV↕•G10↕*and the inverse pattern in *DisR/pV↕*. **C** improvement of *pV*↕ discriminability by ROC&*P_DisR* analysis (red curve) compared to the conventional ROC analysis (black). **D** improvement of *pV*↓ by ROC&*P_DisR* analysis, same format as C; note much larger improvement than obtained for *pV↕*

### Data processing

Each SIFS was characterised by a vector** S** with amplitude A and spatial orientation α derived from the pre- and post-saccadic positions of its horizontal and vertical displacement components captured by the hairline markers. The patterns formed by each pair of consecutive SIFS, i.e. [**S**_n_, **S**_n+1_], [**S**_n+1_, **S**_n+2_] and so on (n, serial number of SIFS) were classified as staircase if the angular difference between the two SIFS of a pair differed by less than 90° cw or ccw and as back-and-forth (BAF) when this difference was larger. STC patterns were characterised quantitatively by the vector average of **S**_n_ and **S**_n+1_, BAF patterns by the vector average of **S**_n_ and −**S**_n+1_ (cf. Figure 1F in Becker et al. 2023). The latter were classified as paired back-and forth patterns (PBF) if their constituent SIFS had ‘similar’ amplitudes (0.8 < A_n+1_/A_n_ < 1.25) and 'similar' motion planes (|α_1_–α_2_|< 22.5°) and as *unpaired* ones otherwise. PBF patterns are essentially analogues of SWJ, except that (1) there is no restriction on the interval between consecutive SIFS, and that (2) not only patterns starting with a SIFS away from the target were classified as PBF patterns, but also those starting with a SIFS returning towards the target if the similarity conditions are met. This implies that the trailing SIFS of a PBF pattern and the following SIFS could also form a PBF pattern.

The results of the fixation task were summarised in terms of the group medians of the vectorial pattern amplitudes (*Amp*), the frequency of pattern occurrence during the fixation period and the displacement rate (*DisR*), i.e. the quotient of the sum of the vectorial amplitudes and the fixation time. These parameters were calculated for of all patterns taken together (yielding global values) as well as separately for PBF patterns.

To quantify the reactions of subjects to target steps in the two VGRS tasks, we determined the peak velocity *pV* of their primary saccades and the gain of these saccades in responses to target steps of 10° (*G10*); the velocity measure *pV* was obtained by first averaging the peak velocities of the evoked saccades separately for each target step size and then adopting the maximum of these averages (cf. section Limitations below).

### Statistics

IBM SPSS^©^ version 28 was used for all statistical analyses. With one exception, the SIFS parameters of controls and patients were not normally distributed (Shapiro–Wilk test). Mann–Whitney U tests were therefore used to compare the two groups. Of the VGRS parameters, about half allowed t-tests for group comparisons while the other half required Mann–Whitney U tests. Similarly, paired t-tests or Wilcoxon tests were used for comparisons within groups. To examine correlations, Spearman's rank correlation coefficient rho was calculated.

For all tests, two-sided error probabilities p < 10^–2^ were considered significant. Given the exploratory nature of our study with many comparisons and correlations, p-values were not adjusted for multiple testing. Only three levels of significance were distinguished (< 10^–2^, < 10^–3^ and < 10^–4^), with the numerous cases of p ≤ 10^–5^ and better being subsumed under 10^–4^.

### ROC analyses

To examine the discriminative power of the various SIFS and VGRS parameters, we obtained their ROC curves. For each parameter, the sensitivity and the specificity at the following six cut-off points on these curves were noted (Fig. 1A): (1) The point of *parity* between sensitivity and specificity; cut-off at this point results in an equal number of false positives and false negatives; the term 1-specificity at this point is a metric of the degree of overlap between the distributions of the discrimination parameter of patients and controls (“overlap index”). (2) The point where Youden's index J reaches its maximum (maxJ); cut-off at this point is supposed to provide an optimal separation between controls and patients (Habibzadeh et al. 2016; Perkins and Schisterman 2006; Youden 1950). However, in a clinical setting, examiners are not interested in statistical optimality but want to be as certain as possible that a subject is affected by PSP (high specificity goal), or they may not want to exclude the possibility that a subject may be affected by PSP (high sensitivity goal). Therefore, we considered primarily four clinically more important points on the curve where, for a given level of specificity (either 90% or 100%), the sensitivity reaches its maximum value, or vice versa, where, for a given level of sensitivity, the specificity reaches its maximum value. These four points are referred to as Spc90, Spc100, Sns90 and Sns100, respectively, and are collectively noted as 90% or 100% points (Fig. 1A). The corresponding best sensitivity values are noted as Sns(Spc90) and Sns(Spc100), and the best specificity values as Spc(Sns90) and Spc(Sns100). As a measure of the trade-off between specificity and sensitivity at these points, we calculated the geometric mean (GM) of specificity and sensitivity. To summarise the discriminative power of VGRS and SIFS parameters, the areas under the ROC curves (AuC; range 0.5 to 1.0) were determined.

## Results

### Fixational and reactive saccades

Based on the results of exploratory ROC analyses, the four best discriminating parameters from the SIFS and VGRS domains, respectively, were retained for further examination (Table 2). All SIFS parameters of patients were significantly larger than the homologues of controls (p < 10^–4^; Table 2A); this was also true for the parameters not shown in Table 2A except for pattern frequency (p < 10^–2^). Conversely, all VGRS parameters were significantly smaller than those of controls (all p < 10^–4^). None of the patients’ SIFS or VGRS parameters correlated significantly with their Unified Parkinson's Disease Rating Scale (UPDRS >< SIFS: p > 0.378, rho = [−0.129 0.002]; UPDRS >< VGRS: p > 0.191, rho = [−0.207 −0.047]). There were also no significant correlations with DD, the duration of the patients' disease (DD >< SIFS: p > 0.188, rho = [−0.193 −0,019]; DD >< VGRS: p > 0.407, rho = [−0,081 0.122]).

**Table 2:** Medians (**A**) and ROC results (**B**) of the four best performing discrimination parameters of the *SIFS and VGRS domains*, respectively, ordered within domains according to their AuC

A		SIFS parameters				VGRS parameters			
		*P_DisR* [°/s]	*Amp* [°]	*DisR* [°/s]	*P_Amp* [°]	*pV↑* [°/s]	*pV↕* [°/s]	*G10↕*	*G10↓*
Controls	Median	0.07	0.35	0.36	0.40	422	419	0.94	1.01
	90% range	0–0.71	0.12–0.98	0.09–2.58	0–1.12	349–532	309–532	0.74–1.05	0.77–1.17
Patients	Median	0.56^c^	0.92^c^	1.68^c^	1.10^c^	219^c^	211^c^	0.50^c^	0.59^c^
	90% range	0.13–2.7	0.34–2.78	0.59–6.41	0.40–2.73	73–407	86–408	0.26–0.97	73–407

B		SIFs parameters				VGRS parameters			
		*P_DisR*	*Amp*	*DisR*	*P_Amp*	*pV↑*	*pV↕*	*G10↕*	*G10↓*
ROC analysis	AuC	0.916	0.891	0.885	0.884	0.958	0.949	0.904	0.902
	Overlap	0.16	0.20	0.20	0.22	0.12	0.12	0.18	0.16
	Sns(Spc90)	0.74	0.66	0.62	0.54	0.88	0.86	0.80	0.80
	Spc(Sns90)	0.80	0.80	0.64	0.72	0.88	0.88	0.72	0.56

Overlap, 1-specificity at the parity point; Sns(Spc90), *sensitivity* at the 90% specificity point; Spc(Sns90), *specificity* at the 90% sensitivity point (see text for definitions); ^**c**^, difference controls *vs*. patients p < 10^–4^

Significant left–right differences of VGRS velocity or gain occurred in neither group. Accordingly, we considered only left–right averages. A significant up-down difference occurred in the control group (*G10*↓ larger). In addition to up-down averages, we therefore examined the upward and downward directions separately. In both controls and patients, vertical saccades were slower and had smaller gain than horizontal ones (all p < 10^–3^ except *G10*). All correlations between the parameters of vertical saccades, whether homodirectional or heterodirectional, reached significant values (all p < 10^–4^) in both controls and patients: *pV*_i_ >< *pV*_j_, rho = [0.790 0.942]; *G10*_i_ >< *G10*_j_, rho = [0,604 0.910] and *pV*_i_ >< *G10*_j_, rho = [0.526 0.790], where i and j denote one of the directions ↑, ↓ or ↕.

Almost no significant correlations occurred between VGRS and SIFS parameters. In the control group, 68 of the 72 VGRS-SIFS relationships (12 VGRS >< 6 SIFS parameters) were not significant (median rho 0.384, error probability p = [0.011 0.992]), with rho positive in 44 cases (median, 0.124) and negative in 24 cases (median, −0.068). Corresponding figures for the PSP group were 70 non-significant relationships (p = [0.011 0.931], median, 0.094), with rho positive in 3 cases (median, 0.180) and negative in 67 cases (median, -0.240). Thus, although not significant, there was a trend in patients for larger and more frequent SIFS to be associated with smaller and slower saccades which is consistent with the notion that the two domains change in an antiparallel sense with disease severity.

### ROC analysis of SIFS and VGRS parameters

Table 2B compares the ROC results of the four best performing parameters from the SIFS and VGRS domains, respectively, in terms of the area under the curve (AuC), the overlap index and the sensitivity and specificity achieved at the Spc90 and Sns90 points. In terms of these characteristics, the displacement rate of paired PBF patterns (*P_DisR*) was the best-discriminating SIFS parameter. Among the VGRS parameters, the upward velocity *pV↑* was the best discriminator, closely followed by the up-down average *pV↕*. Surprisingly, however, the downward velocity *pV↓* (not listed in Table 2) performed much worse than the up-down average *pV↕* (cf. Figure 1 C and D). In contrast, the downward gain (*G10*↓) was one of the two best discriminating gain parameters.

### Improving discrimination by combining parameters

Parameters that are not closely correlated carry at least partially different information, so that their combination can improve discrimination in certain cases. Like Quattrone and colleagues (2022), we considered multiplicative combinations of peak velocity and gain measures as discrimination parameters, namely the homodirectional velocity-gain products *pV*_i_*•G10*_i_ (i = ↑, ↓, ↕) and the heterodirectional combination *pV↑•G10↓*. Furthermore, we examined *pV↕*•*G10↕*)/(*pV ↔ •G10 ↔*), the quotient of the vertical and the horizontal velocity-gain products used by Herwig (2021) and combinations of *pV↕* or (*pV↕•G10↕*) with both the global SIFS displacement rate (*DisR*) and the displacement rate of the paired PBF patterns (*P_DisR*) in the form of the ratios *DisR*/*pV↕, P_DisR*/*pV↕*, *DisR*/(*pV↕*•*G10↕*) and *P_DisR*/(*pV↕•G10↕*).

**Fig. 2:**
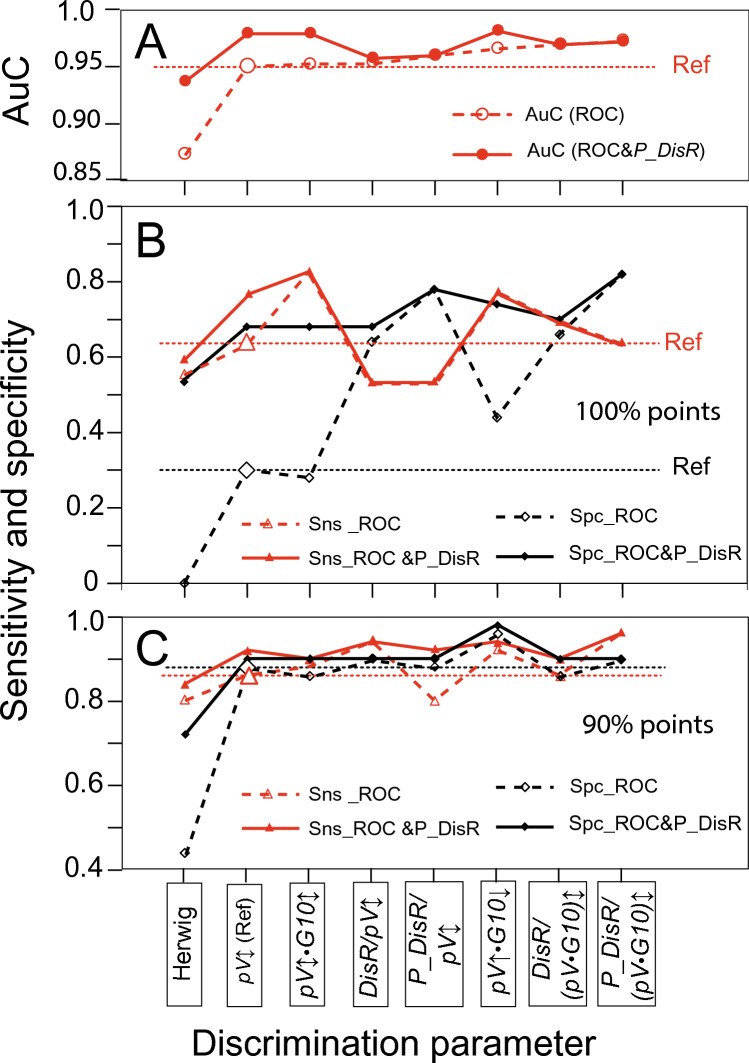
Effect of parameter combinations and of ROC&P_DisR analyses on the sensitivity and the specificity at the 100% and the 90% points. Same data as Table 3 but parameters sorted in ascending order along abscissa according to the AUC of the conventional ROC analyses. Dashed curves, results of conventional ROC analyses; continuous curves, results of ROC&*P_DisR* analyses. Dotted horizontal lines, results of reference parameter *pV↕*. **A** area under ROC curves. **B** comparison of conventional ROC and ROC&*P_DisR* analyses. **Sns_ROC** and **Sns_ROC,** *specificity* at Spc100 and *sensitivity* at Sns100, respectively, obtained with conventional ROC analyses; **Sns_ROC&P_DisR** and **Spc_ROC&P_DisR,** corresponding values from ROC&*P_DisR* analyses. **C** Sns(Spc90) and Spc(Sns90) results, same presentation as in B. See top of text for resolution of acronyms on abscissa

Table 3 A&C and the *dashed* curves in Fig. 2 compare the performance of the two best performing *pV•G10* products and of the combinations including SIFS parameters against *pV↕* as a reference in terms of their AuC, their overlap indices and their sensitivities and specificities at the 100% and 90% points. All composite parameters except Herwig's index had greater AuC than *pV↕*, although some differences were small. Among the multiplicative combinations, AuC was largest in *pV↑*•*G10↓* but was surpassed by the AuC of *P_DisR*/(*pV↕•G10↕*). Except in the case of Herwig’s index, AuC was a poor predictor of the performance at the 100% points. The *sensitivity* at Spc100 did not reflect the increase in AuC along the abscissa in Fig. 2A but varied unpredictably around the reference level set by *pV*↕ Fig. 2B), being substantially enhanced in the two *pV*⋅*G10* combinations (increase relative to reference: Δ = 0.14 and 0.20) but reduced or unchanged in combinations with SIFS parameters. In contrast, the *specificity* at Sns100 increased in all composites (Δ = [0.14 0.52]), except in *pV↕•G10↕* and in Herwig’s index (zero sensitivity).

**Table 3:** Improving discriminability using parameter combinations and ROC&P_DisR analyses

		pV↕	pV↕G10↕	pV↑G10↓	Herwig	DisR/pV↕	P_DisR/pV↕	DisR/(pV↕•G10↕)	P_DisR/(pV↕•G10↕)
**A**	AUC	0.949	0.952	0.964	0.872	0.952	0.958	0.968	0.971
Without &P_DisR	OvInd	0.12	0.12	0.08	0.18	0.12	0.10	0.12	0.10
	Sns(Spc100)	0.64	0.84	0.78	0.56	0.54	0.54	0.70	0.64
	Cut-off	*262°/s*	*241°/s*	*234°/s*	*0.48*	*7.9⋅10*^*–3*^	*26.5⋅10*^*–4*^	*8.1⋅10*^*–3*^	*30.2⋅10*^*–4*^
	Spc(Sns100)	0.30	0.28	0.44	0.00	0.64	0.78	0.66	0.82
	Cut-off	*451°/s*	*430°/*	*445°/*	*6.24*	*1.4⋅10*^*–3*^	*3.6⋅10*^*–4*^	*1.5⋅10*^*–3*^	*5.1⋅10*^*–4*^
**B**	AUC	0.978	0.978	0.980	0.936	0.956	0.958	0.968	0.971
With &P_DisR	OvInd	0.08	0.10	0.08	0.18	0.10	0.10	0.10	0.10
	Sns(Spc100)	0.76	0.84	0.78	0.60	0.54	0.54	0.70	0.64
	Cut-off	*351°/*	*291°/*	*326°/*	*0,72*	*3,72⋅10*^*–3*^	*7,18⋅10*^*–4*^	*4,41⋅10*^*–3*^	*7,12⋅10*^*–4*^
	Spc(Sns100)	0.68	0.68	0.74	0.54	0.68	0.78	0.70	0.82
	Cut-off	*358°/*	*320°/*	*297°/*	*0.88*	*3.12⋅10*^*–3*^	*8.50⋅10*^*–4*^	*3.93⋅10*^*–3*^	*9.95⋅10*^*–4*^
**C**	Sns(Spc90)	0.86	0.88	0.92	0.80	0.80	0.94	0.86	0.96
Without &P_DisR	Cut-off	*352*°/s	*291*°/s	*326*°/s	*0.72*	*3.72⋅10*^*–3*^	*7.18⋅10*^*–4*^	*4.41*	*7.12⋅10*^*–4*^
	Spc(Sns90)	0.88	0.86	0.96	0.44	0.88	0.90	0.86	0.90
	Cut-off	*358°/s*	*320°/s*	*297°/s*	*0.88*	*3.21⋅10*^*–3*^	*8.50⋅10*^*–4*^	*3.93*	*9.93⋅10*^*–4*^
**D**	Sns(Spc90)	0,92	0,90	0,94	0,84	0,92	0,94	0,90	0,96
With &P_DisR	Cut-off	392°/s	*342°/s*	*377°/s*	*0,76*	*3,02⋅10*^*–3*^	*7,18⋅10*^*–4*^	*4,01⋅10*^*–3*^	*7,12⋅10*^*–4*^
	Spc(Sns90)	0.90	0.90	0.98	0.72	0.90	0.90	0.90	0.90
	Cut-off	*392°/s*	*342°/s*	*297°/s*	*0,88*	*3.12⋅10*^*–3*^	*8.5⋅10*^*–4*^	*4.01⋅10*^*–3*^	*9.95⋅10*^*–4*^

**A**, AuC values and specificities and sensitivities at the 100% points obtained with conventional ROC analyses in *pV*↕ and in combinations of *pV↕* with other VGRS parameters and SIFS parameters, respectively. **B**, AuC values and specificities and sensitivities obtained at the 100% points from ROC&*P_DisrR* analyses. **C** and** D**; analogues of A and B at the 90% points. OvInd, overlap index (= 1-specificity at the parity point). For resolution of parameter acronyms in header see text

The results at the 90% points (Fig. 2C) gave, as expected, larger sensitivities and specificities compared to the 100% points. The sensitivity at Spc90 again did not reflect the corresponding AuC values well; it reached a maximum of 0.96 (Δ = 0.10) in *P_DisR/(pVG10)↕.* The specificity at Sns90 showed a more systematic, but still not significant, dependence on AuC and reached its maximum in *pV↑•G10↓*, also with a value of 0.96 (Δ = 0.08). These two maxima gave the best sensitivity–specificity trade-offs (GM = 0.93) of all parameters and ROC curve points examined here.

### Improving discrimination by examination of SIFS parameters

**Fig. 3:**
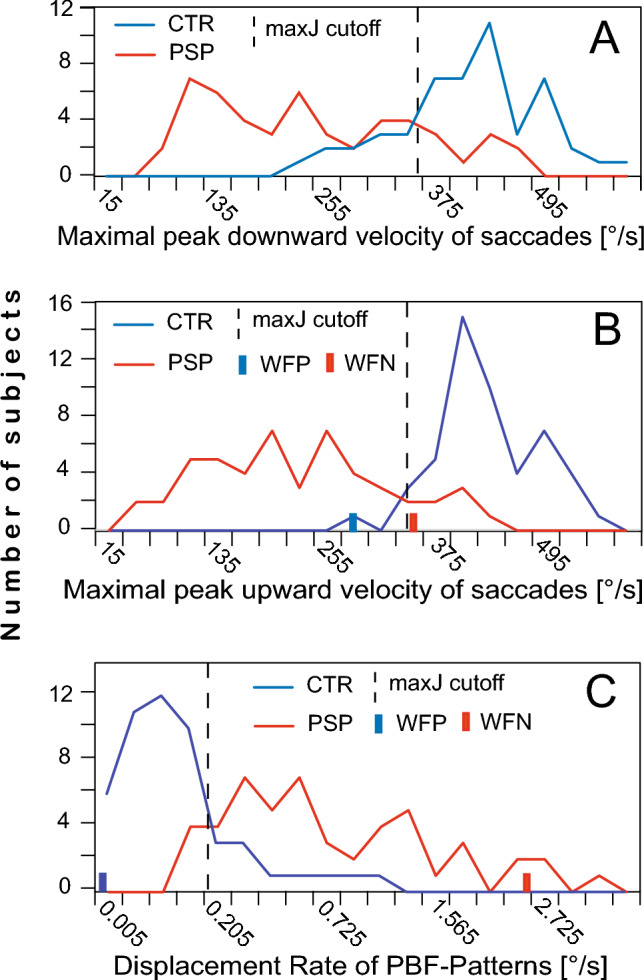
Frequency distributions in controls (blue traces) and patients (red traces) of the downward (**A**) and upward (**B**) maximal peak velocities of visually guided reactive saccades (*pV↓* and *pV↑*, respectively) and of the displacement rates (**C**) resulting from small involuntary fixation saccades forming square-wave like PBF patterns (*P_DisR*). Dashed vertical lines mark cut-off values maximising Youden’s index J. Bars represent a control subject (blue) wrongly classified false positive (WFP) by *pV↑* (panel B) and a patient (red) wrongly classified false negative (WFN) whose misclassifications could be corrected by considering their position in the distribution of the *P_DisR* values (panel C; note that data in C are square root transformed for better representation of the very small values of controls). Red and blue segments of abscissae mark ranges where the distributions from patients and controls do not overlap

As shown in Fig. 3C, the frequency distributions of the *P_DisR* values of the two groups overlap only partially. Very low values occur only in controls and very high values only in patients. On the ROC curve of *P_DisR* (Fig. 1A), these ranges correspond to the curve sections with 100% sensitivity and 100% specificity, respectively. This suggests that subjects of unknown affiliation, whose *P_DisR* values fall within one of these overlap-free ranges, could be classified a priori with low error likelihood as either controls or patients, irrespective of their classification by VGRS parameters. As an example, the bars on the abscissa in Fig. 3B represent a control subject (blue) and a patient (red) who were misclassified as patient and control, respectively based on the cut-off of their upward eye velocity (*pV↑*) at the point maximising Youden’s index but were correctly identified by considering their *P_DisR* values (Fig. 3C). To systematically approach the use of extreme values of *P_DisR* for the improvement of discrimination, we defined, on the basis of the distributions in Fig. 3C, values above 1.5°/s as highly unlikely for controls and values below 0.08°/s as unlikely for patients. Accordingly, subjects with *P_DisR* > 1.5°/s or < 0.08°/s were a priori considered patients (N = 9) or controls (N = 28) and removed from the sample. The reduced sample was subjected to a conventional ROC analysis, but at each cut-off level the number of controls and patients identified a priori by *P_DisR* was added to the count of true negatives and true positives, respectively. Figure 1 C&D compare ROC curves modified in this way (red) with the conventional ROC curves of the same parameter (black). In both examples, the specificity and sensitivity values along the modified ROC curves (hereafter referred to as ROC&*P_DisR* curves) are better than or equal to those of the conventional curves. The comparison of panels B and C illustrates a general trend: poorly discriminating parameters (here *pV↓*) benefit more from ROC&P_DisR analyses than better discriminating parameters (here *pV↕*).

Table 3 B&D and the continuous curves in Fig. 2 show the results obtained with ROC&*P_DisR* analyses (Δ quotations now refer to the conventional ROC results of each parameter). The AuC of the reference *pV↕*, Herwig’s index and the multiplicative combination of vertical velocity and gain (*pV↕•G10↕*) increased considerably (Δ = [0.026 0.064)], and to a lesser degree also the AUC of the heterodirectional combination *pV↑•G10↓*, whereas the AuC of combinations with SIFS parameters did not change (Δ = [0.000 0.004]). All sensitivities and specificities were better than or at least equal to the corresponding values of the conventional ROC analysis. While Sns(Spc100) improved only in *pV↕* (Δ = 0.12) and Herwig's index (Δ = 0.04), Spc(Sns100) increased considerably (Δ = [0.28 0.54]) in all parameters free of SIFS components but only minimally or not at all in combinations with SIFS parameters (Table 3B). The resulting curve in Fig. 2B (continuous black) was clearly less jagged than that of the conventional ROC analyses (dashed). At the 90% points, where the results of conventional ROC analyses were much closer to 1.00 than at the 100% points, the ROC&*P_DisR* analyses (Table 3D) gave only small increases in sensitivity and specificity (Δ ≤ 0.06) and none in composites with *P_DisR*; exceptions were the Sns(Spc90) of *P_DisR/pV↕* (Δ = 0.12) and the Spc(Sns90) of Herwig's index (Δ = 0.28); however, even with this increase, Herwig's index did not reach the level of the conventional ROC analyses of the other parameters Finally, at the Sns90 point, the ROC&*P_DisR* analysis gave the best specificity-sensitivity trade-offs of all analyses, with a specificity of 0.98 in *pV↑•G10↓* and thus a geometric mean (GM) of 0.94, closely followed by *P_DisR/(pVG10)↕* which achieved a sensitivity of 0.96 at the Spc90 point (GM = 0.93).

### Effect of disease duration

With a view to a possible application of the present methods to the particularly relevant challenge of differentiating PSP from PD in their early stages, we investigated whether and to what extent our results differ between patients with short and long disease durations (DD). We formed groups of 25 patients each, one with short DD (23 cases with DD < 3 years, plus 2 randomly selected cases with 3 years) and one with long DD (25 cases with DD > 2 years). Two mutually exclusive groups of 25 controls each served as counterparts. The four possible combinations of short and long DD groups resulted in two S- and two L-samples, respectively, which were subjected to ROC analyses. In the L-samples, the AUC of the examined parameters was slightly smaller, in the S-samples larger compared to the full sample. To check whether the result of ROC&*P_DisR* analyses would depend on DD, we noted for each S- and L-sample the the number of controls and patients falling into the respective overlap-free regions of their *P_DisR* distributions. The two thirds of them with the smallest (controls) and largest (patients) *P_DisR* values then were taken as representative of the achievable correct a priori identifications. The two S-samples allowed 6 and 4 a priori identifications of PSP, the two L-samples 4 and 5, respectively; corresponding figures for the identification of control subjects were 10 and 13 (S-samples) and 9 and 13 (L-samples). The corresponding limits of *P_DisR* for identifying controls were 0.055 and 0.083°/s^2^ in S- and L-samples, respectively, those for identifying patients 1.44 and 1.32°/s^2^. The above figures provide no hint at a dependence of the a priori identifications on DD.

Consistent with the above findings, the average AUC values of the four combinations of SIFS parameters with VGRS parameters* (*i.e. *pV*↕ or *pV↕*⋅*G10*↕ with *DisR* or *P_DisR*) also showed no influence of DD on average (short, 0.961 [0.940 0.976]; long, 0.963 [0.942 0.984]), being similar to those of the whole sample where the corresponding AUC values averaged 0.963 (cf. Table 3B). They also showed similar *improvements* over their reference, the AUC of vertical velocity in the respective samples, with means of 0.015 [−0.008 0.031] (S-samples) and 0.011 [−0.18 0.040] (L-samples).

On average, the sensitivities and specificities of the discrimination parameters were similar in the two S-samples; the difference averaged over the seven parameters listed in Table 3 (excluding Herwigs’ index) and over the Spc100 and Sns100 points was -0.03, masking a considerable scatter ([−0.24 0.36]) between the individual parameters. The corresponding values at the 90% points were 0.03 and [−0.52 0.32]. The largest differences between the two samples occurred in the velocity-gain combinations. However, for further analyses the mean values of the two S-samples and likewise of the two L-samples were determined.

In contrast to the similar AUC values of combinations with SIFS parameters in the S- and L-samples, the AUC values of the vertical velocity-gain combinations were smaller for S-samples than for L-samples (mean 0. 945 versus 0. 965). These differences in AUC did not correspond to the differences in discriminatory power at the 100% points, as the S-samples performed equally or better than both the L-samples and the total sample in most respects rather than worse. For example, the averages of the reference parameter (*pV*↕) for the sensitivity at Spc100 and the specificity at Sns100 were higher by 0.06 and 0.22, respectively, in the S-samples than in the total sample.

In the following we focus on the improvements or deteriorations (Δ) of the composite parameters relative to their reference values in the S-samples since Δ decides whether it is worthwhile to use a composite rather than *pV*↕ alone. For Sns(Spc100) both *velocity-gain combinations* (*pV*↕*G10*↕, *pV*↑*•G10↓*) showed a clear improvement with Δ = 0.12 and 0.10, whereas Spc(Sns100) improved only in the heterodirectional combination *pV*↑⋅*G10*↓ (Δ = 0.22) but deteriorated in *pV*↕*•G10*↕ (Δ = -0.12). *Combinations of pV↕ with the SIFS parameters DisR* and *P_DisR* gave only negative Δ values ([−0.12 −0.04]) for Sns(Spc100) but large improvements for Spc(Sns100) with Δ = [0.12 0.38]. However, at the 90% points the situation was partially reversed: here, the reference parameter *pV*↕ of S-samples had lower values than in the total sample (Sns(Spc90): Δ = −0.02; Spc(Sns90): Δ = −0.16) and the sensitivity at Spc90 of the velocity-gain combination *pV*↕*G10*↕ at Spc90 deteriorated relative to its reference (Δ = −0.10) rather than improving as at the 100% point; only the other velocity-gain combination, *pV*↑*G10*↓, showed a minor improvement (Δ = 0.04). The specificity at Sns90 deteriorated with both combinations (Δ = −0.26 and −0.04). The sensitivities at Spc90 of combinations with SIFS parameters improved or deteriorated variably (Δ = [−0.04 0.04]) rather than being all worse than the reference, and their specificities at Sns90 all improved (Δ = [0.14 0.18], with the overall best result being achieved by *P_DisR*/*pV*↕*G10*↕. In terms of the compromise index GM, most parameters, including the reference *pV↕*, performed better at the 90% than at the 100% points of the S-samples.

## Discussion

Using ROC analyses, we investigated different methods to improve the discrimination between patients and healthy subjects beyond what can be achieved by using the vertical velocity of reactive saccades (*pV*↕) as the discrimination parameter. Improvements were gauged by several indicators, namely the sensitivities and specificities reached at standard points of the ROC curves and by the area under the curves (AUC); in this context, it is worth noting the caveat that AUC, which is a global measure, is often a poor predictor of the characteristics at specific points of the ROC curve. In a sample of 50 patients and 50 controls, three approaches were successful, i.e. (1) combining vertical velocity with the gain of vertical saccades or with (2) displacement rate measures (*Dis_R*, *P_DisR*) of fixational saccades into a composite discrimination parameter and (3) using ROC&*P_DisR* curves, obtained by a modified ROC analysis incorporating a priori information about the group affiliation of subjects provided by the displacement rate of *P_DisR*. The improvements obtained depended on the parameter combinations and on the specificity and sensitivity levels considered (100 or 90%).

Although not addressed in the current study, some of these methods might also be applicable to the clinically relevant challenge of deciding whether a patient has PSP or PD to potentially improve the discrimination compared to using vertical velocity parameters alone. PD patients have about the same vertical saccade velocity as healthy subjects (Pinkhardt et al. 2008, 2011; Quattrone et al. 2022) and the same velocity-gain products (Quattrone *ibid*); accordingly, vertical saccade velocity discriminates between PSP and PD patients almost as well as between PSP and controls, and velocity-gain products potentially enhance this discrimination. The SIFS displacement rate of PD patients is likely to be somewhat higher than that of controls. Reportedly, the two factors that co-determine the displacement rate of the paired PBF patterns of SIFS (*P_DisR*), SIFS amplitude and frequency, are higher in PD than in controls, on average by factors of approximately 1.15 to 1.35 (amplitude) and 0.85 to 2.0 (frequency) (Lage et al. 2024; Otero-Millan et al. 2013; Pinnock et al. 2010). Therefore, *P_DisR* would contribute less to the discrimination between PSP and PD compared to that between PSP and controls. However, the extent to which these larger amplitudes reduce the contribution of *P_DisR* to the discrimination between PSP and PD also depends on the distribution of *P_DisR* in PD patients which is the critical factor for the ROC&*P_DisR* analyses.

As noted above, the clinical distinction between PSP and PD is particularly difficult in the early stages of the disease. Our separate analyses of cases with short and long disease duration (DD) caution that velocity-gain combinations may offer no advantage as discriminators over velocity alone at 90% points in cases with short DD. Combinations with SIFS parameters were also not helpful in these cases except for clear improvements in specificity at 90% sensitivity. However, the a priori identification of a subject’s affiliation on the basis of the displacement rate *P_DisR* does not depend on DD. Therefore, the performance of the ROC&*P_DisR* method is not expected to be reduced in early-stage patients. Before discussing these issues in more detail, we now first briefly consider the central values of, and the interrelations between, the VGRS and SIFS parameters underlying our study.

### SIFS parameters

The median values of the SIFS parameters were very similar to those reported previously (Becker et al. 2023) and are consistent with the results of other studies (Donaghy et al. 2009; McGivern and Gibson 2006; Nij Bijvank et al. 2019; Otero-Millan et al. 2011; 2013; Pinnock et al. 2010). By the same token, the significant differences between patients and controls (larger amplitudes and displacement rates in patients) were reproduced. A particularly large difference between patients and controls occurs in *P_DisR*, the displacement rate resulting from SIFS forming PBF patterns, our analogue of SWJ. The main reason for this finding is the larger amplitude of fixational saccades in patients, which increases the probability of occurrence of paired back-and-forth fixational saccades (Becker et al. 2023; Otero-Millan et al. 2011, 2013); this results in a disproportionately large influence of SIFS amplitude on the magnitude of *P_DisR* in patients. On the other hand, the fixational saccades of some controls were so small that no PBF patterns could form and no *P_DisR* could arise. Taken together, these effects increase the contrast between patients and controls and provide the basis for the almost sure classification of some subjects as patients or healthy individuals by examining *P_DisR.* According to its principle, this method is largely independent of the patients' disease duration (DD). Although *P_DisR* may increase slightly with DD, the lower limit of this distribution set by patients with short DD remains the same, as does the upper limit of the overlap-free part of the *P_DisR* distribution of controls. Therefore, the number of non-PSP subjects (healthy subjects and possibly PD patients) that can be identified a priori does not change when only early stages of PSP are considered. In contrast, the a priori identification of patients with short DD could be limited if the upper limit of their distribution protrudes less beyond the upper limit of the non-PSP subjects. However, the present data showed no such effect, as the number of controls and patients detected did not differ between the S- and L-samples.

### VGRS parameters

Our observation that the vertical saccades of the PSP group exhibit no significant up-down differences is consistent with Chen et al. (2010) and Pinkhardt et al. (2008), but contrasts with qualitative data from other studies that suggest faster and larger downward movements (Quattrone et al. 2022; Herwig et al. 2021). According to Chen (*ibid*), and as found here, control subjects do not show an up-down difference either. However, others have reported higher velocities and greater gain in the downward direction (Bonnet et al. 2013). Our choice of the up-down average of the velocity (*pV*↕) as the reference for the study of improvements is a trade-off accounting for these discrepancies.

### ROC analyses of VGRS and SIFS parameters

Several recent studies on the use of saccadic eye movement recordings to discriminate PSP patients from healthy subjects or PD patients have used ROC analyses (Herwig et al. 2021; Marx et al. 2012; Quattrone et al. 2022; Wunderlich et al. 2021) but have only focused on a single point with high specificity. In some studies, this point resulted from a cut-off chosen to have a predefined z-score distance (e.g. -2.5) from the sample mean of the discrimination parameter in controls. However, because the frequency distributions of different parameters differ from each other, the resulting points on the ROC curve also differ. For example, Quattrone et al. (*ibid*) landed in this way on the ROC curve of peak vertical velocity at a specificity of 0.991 and a sensitivity of 0.549 (GM = 0.738) whereas their velocity gain composite led to a specificity of 0.947 and a sensitivity of 0.843 (GM = 0.893). Whether the better trade-off between specificity and sensitivity obtained with the velocity gain composite could also have been achieved by lowering the specificity on the ROC curve of the velocity cannot be answered in this way. Also, comparisons with other studies are difficult or impossible if the results do not refer to standard ROC points. Here we choose Spc100 and Spc90 to cover the range that is clinically most relevant for distinguishing PSP patients from controls or patients with another disease. As there may be problems where one might want to identify as many patients as possible with minimal by-catch of non-target subjects, the ‘mirror points’ Sns100 and Sns90 are also of interest. Moreover, with values of 0.96 or 0.98 in two cases, the specificity at Sns90 points gave the best trade-offs between specificity and sensitivity.

A puzzling observation at first glance is the much lower discrimination performance of the peak downward saccade velocity (*pV*↓) compared to the upward velocity (*pV*↑) in our sample (Fig. 2C and D). Both controls and patients showed no significant up-down differences in saccade velocity that could explain this difference, nor was there any up-down asymmetry in the velocity differences between the two groups. The real cause of this discrepancy is the fact that *pV↓* and *pV↑* both had different frequency distributions in controls and patients and yet similar central values. As a result, the overlap between the velocities of controls and patients was downwards considerably greater than upwards (Fig. 3A and B). To critically assess this observation, it should be noted that the jagged nature of the curves in Fig. 3A and B suggests that the distributions are strongly influenced by random effects due to the limited sample size of 50 subjects per group. It is therefore premature to recommend upward velocity as the best discriminator. As a compromise, we have therefore chosen the up-down average (*pV*↕) as the reference for evaluating improvements.

As a metric for the comparison of studies, AuC values are often quoted. However, it is important to remember that AuC is a global measure that may have little relevance at specific points where performance depends on details of how the distributions of the discrimination parameters of patients and controls overlap. For example, Wunderlich et al. (2021) report a significantly lower AuC for downward peak velocity compared to upward, but almost identical sensitivities associated with a specificity of 0.955 in both directions. Another example is provided by the velocity-gain combinations which despite a larger AUC had a lower sensitivity at Spc100 in L-samples compared to S-samples; this apparent paradox is due to the course of the ROC curves in their high specificity region (not shown).

With vertical peak saccade velocity *pV*↕ as a discriminator, we have obtained an AuC of 0.949. This is inferior to the AuC of 1.00 obtained by Marx et al. (2012) using vertical velocity to discriminate between 10 PSP and 11 PD patients. This perfect discrimination may be due to a sampling bias, as the PSP group only included patients who had a vertical eye movement impairment on *clinical* examination; this visual inspection may have excluded patients with smaller velocity reductions, especially as the velocity of the controls was already low because only small saccades were examined. The AuC of vertical peak velocity obtained by Wunderlich et al. (2021) was smaller than that of our parameter *pV*↕, and the sensitivity associated with the specificity of 0.96 resulting from the chosen cut-off was slightly lower than that of our *pV*↕ at Spc100 (0.57 *vs* 0.64). The same holds for the sensitivity of 0.55 reported by Quattrone et al. (2022) for a specificity of 0.99. Thus, in the present study, the discriminative power of *pV↕* was better than in the above studies except for the extreme and possibly biased result of Marx et al. (2012). There is no obvious methodological difference that could explain this better performance of the present *pV↕*, which is probably a chance result; in all studies, patients were recruited according to the MDS criteria for PSP (Höglinger et al. 2017) and the recording equipment would only matter if it introduced non-linearities.

However, all data on the discrimination performance of *pV*↕ and its combinations cited above, including ours, were obtained with samples containing patients with long disease duration (in all reports median DD ≥ 3 years). The contrasting discrimination behaviour of *pV*↕ in S-samples at the 100% points (improvement compared to the total sample) and the 90% points (deterioration) makes it difficult to predict its performance in the case of patients in the early stages of the disease. However, the performance at the 90% points is likely to be more representative of the early-stage results as it is less dependent on random outliers of the discrimination parameter than performance at the 100% points. Therefore, early-stage patients might be harder to detect than those with longer disease duration. At any rate, it is a puzzle why the ROC results from S- and L-samples differ considerably in many aspects from those of the full sample given that the parameters tested do not vary with DD. Speculatively, smaller sample sizes and changes in the parameter distributions can be cited as possible causes, i.e. ultimately random factors.

According to their ROC curves, parameters based on spontaneous involuntary fixational saccades (SIFS) discriminated patients and controls clearly less well than the velocity and gain parameters of reactive saccades, in keeping with their 3rd order weight in the current MDS diagnostic criteria. Thus, they are not the first choice for separating patients from healthy subjects. However, the displacement rate of paired back-and forth patterns of SIFS (*P_DisR*) could serve as an alternative approach when patients are unable to perform the VGRS task. The only other ROC analysis of SIFS parameters as discriminators between PSP and controls that we are aware of (Otero-Millan et al. 2011) examined SWJ and found an AuC of about 0.88 for the amplitude of SWJ, a value similar to that of our equivalence *Dis_R* (0.885), the *global* displacement rate of SIFS.

Note that SIFS have since long also been known as “saccadic intrusions”. Therefore, their displacement rates *DisR* and *P_DisR* should not be confounded with the “saccadic intrusion rate” as described by Wunderlich et al. (2021) where this rate was the sum of the corrections for the undershoot of the primary saccade in the VGRS task.

### Combinations of velocity and gain criteria

A prerequisite for improving the discrimination between PSP and controls by combining two parameters is that these parameters are not closely correlated in either group. Otherwise, subjects would be ranked the same way by the ROC analysis regardless of whether the original parameters or their combination were considered. Since ranking is the basis of the ROC analysis, all parameters would then perform identically. The above prerequisite was met in the case of multiplicative velocity-gain combinations (*pVG10*) despite their relatively high correlation coefficients, as there was a mean rank difference (absolute value) between, for example, vertical velocity *pV↕* and gain *G10↕* of 8.5 with a maximum of 31. Of all the composite parameters tested, the two velocity gain combinations showed the greatest improvements in sensitivity at Spc100 compared to vertical velocity alone and were superior also at Spc90. Thus, multiplicative velocity gain combinations offer a benefit, probably by mitigating extremes of one component by more typical values of the other. However, this benefit may be limited or not present in early-stage patients.

Our instantiation of the more complex combination of velocity and gain used by Herwig et al. (2021) gave approximately the same sensitivity (0.80) at Spc90 as the original work (0.77); however, compared to the sensitivity of *pV↕* alone (0.86), this represents a clear deterioration rather than an improvement. Without knowing what the performance of the vertical velocity of Herwig's sample would have been per se, it is difficult to assess whether the index could be advantageous in samples other than ours.

### Combination of SIFS and VGRS parameters

The fact that SIFS parameters such as displacement rates correlated only weakly with the parameters of VGRS but can also discriminate between patients and controls to some extent suggested that combining VGRS and SIFS parameters may also be a way to improve discrimination. This was indeed the case but applied mainly to the specificity at the 100% sensitivity point rather than the more interesting sensitivity at the Spc100 point. This is because the values of the reference parameter (vertical saccade velocity) of the patients scattered far into the range of the control subjects, whereas the scatter of the control subjects reached less into the range of the patients. This asymmetry, which is reflected by the only slow attainment of 100% sensitivity by the ROC curve of the reference *pV*↕ (Fig. 1A, blue curve), is counterbalanced in these combinations by the low scattering of the patients' displacement rate measures (*DisR*, *P_DisR*) into the range of the control subjects, sometimes at the expense of a lower sensitivity at Spc100, though (Fig. 1B, black. One of the VGRS-SIFS combinations, *P_DisR/(pV↕•G10↕)* which profits from the “filtering” effect of *P_DisR*, showed the largest AuC of all parameters studied. The promise associated with this large AuC was fulfilled at the Spc90 point, where the sensitivity (0.96) was significantly higher than that of any other parameter, resulting in the second-best trade-off between specificity and sensitivity (GM = 0.93).

### ROC&P_DisR analysis

The second and more direct way to profit from the displacement rate *P_DisR* are ROC&*P_DisR* analyses. Their principle implies that the result is always better or equal to that obtained without prior knowledge of *P_DisR*, unlike the ROC results of parameter combinations which can be worse in parts than those of their components (Fig. 2B and C). The method rests on the a priori identification of subjects with a high likelihood of belonging to either the patient or the control group according to the magnitude of their *P_DisR*. In our sample of subjects, the *P_DisR* values of a large proportion of the controls (N = 33) fell into a value range in which no patients occurred, and which extended from 0 to about 0.1°/s. Conversely, a smaller fraction of the patients (N = 14) fell into a range where no controls occurred, which extended from 1.2 to 3.6°/s. The limits of these ranges in our sample are random results that may not be representative of the populations at large. Obviously, the likelihood of correctly classifying a person of unknown group membership a priori decreases the closer their *P_DisR* is to one of the critical limits. As a pragmatic approach, we considered the five controls closest to the upper boundary of the free-of-patients range as subjects of unknown affiliation and similarly the three subjects closest to the lower boundary of the free-of-controls region. This corresponds to heuristic safety margins of 15 and 21%, respectively, to be respected when dealing with persons of unknown group affiliation.

Obviously, only those of the a priori identified patients and controls that escape correct detection by the discrimination parameter under study help to improve discrimination by way of a ROC&*P_DisR* analysis. Accordingly, the lack of improvement by ROC&*P_DisR* analysis of composites including *P_DisR* indicates that these composites have correctly classified all subjects belonging to the overlap-free areas.

### Parameter combinations versus prior examination of P_DisR

Judging from the present sample, when no reliable information on disease duration (DD) is available, the velocity-gain combinations *pV*↕*•G10*↕ and *pV*↑*•G10*↓ provide the best sensitivity if a specificity of 100% is required, whereas most combinations with the SIFS parameters *DisR* or *P_DisR* give no improvement. A ROC&*P_DisR* analysis of all these composite parameters gives no further improvement in sensitivity; it only improves the sensitivity of *pV↕*. However, if a specificity of 90% is acceptable, small improvements in sensitivity are obtained with all parameter combinations, and further small improvements result from a ROC&*P_DisR* analysis in some cases. Both the ROC&*P_DisR* method and the combination of VGRS and SIFS parameters produced greater improvements at the Sns100 points than at the Spc100 points. In the case of the patients with short DD from our S-samples, the partially contradictory results at the 100% and 90% points make a summary judgement difficult. As the latter points are probably more representative of cases with short DD, we refer here to the performance at Spc 90, where only the combinations *pV*↑*G10*↓ and *P_DisR*/(*pV*↕*•G10↕*) led to an increase in sensitivity relative to the reference *pV↕*, whereas at Sns90 the specificity of both velocity gain combinations worsened, but all combinations with SIFS parameters improved. We have shown that an a priori detection of likely patients or controls is also possible in samples with short DD. This is the is the prerequisite for ROC&*P_DisR* analyses; the improvement from such analyses depends on how many of the detected subjects are missed by *pV*↕ or its combinations.

What are the practical implications of our study? As few clinical laboratories currently perform regular quantitative analyses of patients' fixation saccades, only the multiplicative combination of vertical velocity and gain can currently be considered as a possible real-world improvement over velocity alone when the patient's disease duration is uncertain or unknown. Where fixation saccades are at least recorded, it is possible to check whether they are very small or absent which would rule out PSP, or very large which would rule out non-PSP subjects. However, the full benefit of considering fixation saccades can only be realised if *P_DisR*, the rate of displacement of paired back-and-forth (PBF) patterns (or presumably SWJ), can be determined. According to the present data, this method can also be expected to improve the detection of patients in the early stages of the disease compared to vertical saccade velocity as a discrimination parameter.

### Limitations

A main limitation of this study is the uncertainty about how much of the reported differences between the various test parameters and procedures are due to random factors. It is highly unlikely that the prominent peaks and troughs of parameter distributions such as shown in Fig. 3 are representative of patients and controls at large; they are rather due to our limited sample size. Their shapes at the borders of the overlap region between patients and controls determine how the ROC curves approach Spc100 and Sns100 and therefore the sensitivity or specificity at these points (cf. Figure 1B). Nevertheless, several of our results can certainly not be dismissed as coincidental findings. This applies to the improvement in discrimination with velocity and velocity-gain combinations when patients with long disease duration are considered. More importantly, however, the possibility to improve the discrimination by an a priori examination of a subject’s *P_DisR* value definitely also works in early-stage patients. We are confident that the *P_DisR* values of patients will rarely fall within the near-zero range typical of controls, and similarly those of healthy controls into the high-end range typical of patients. What is currently lacking, however, is a reliable delineation of these areas to replace our heuristic approach. Nevertheless, the overall fairly similar limits of these ranges found in our long and short disease duration subsamples lend some credibility to the values reported here.

The limited sample size must also be kept in mind when evaluating the differences found between the various parameters and conditions, since a single subject with an extreme parameter value can account for large differences in sensitivity or specificity close to the 100% points but less so at the 90% points. A similar comment applies to the cut-off values listed in Table 2, which apply to our sample but may not be representative of the whole population. In addition, a major limitation is the lack of autopsy-confirmed diagnoses of PSP.

A fundamental challenge in dealing with the target step responses of some PSP patients is to define which part of their response should be considered the primary saccade. The first saccade following a target step is often very small and indistinguishable from involuntary fixational saccades and can only be identified as such if its latency is atypically short. It is usually followed either by a staircase of several small corrective saccades or by a large corrective movement which could just as well be the first real reaction instead of the preceding small saccade. Hence, every parameter nominally related to the primary saccade risks to assume arbitrary values in such cases. Accepting small SIFS-like saccades as primary risks to exaggerate the differences in gain between patients and controls (which may actually improve the discriminability) and to underestimate the difference in velocity as the velocity of small vertical saccades (≤ 5°) of PSP patients is difficult to distinguish from that of healthy subjects (Averbuch-Heller et al. 2002). This uncertain definition of “primary” also affects the discriminators *pV* and *G10* used here and motivated the procedure described in Methods. For future studies, we would record the velocity of the largest saccade of each reaction and search among the target step size categories for the highest average velocity of these saccades. Similarly, the largest saccades of this same category could also be used to define a gain referring either to the fixation error existing at saccade onset or to the target step magnitude.

Finally, the interactive identification of the steady-state displacement of SIFS used here is impracticable under routine conditions. However, it could be replaced by dedicated algorithms that could also answer the question of whether a displacement measurement including the dynamic overshoot of SIFS leads to a more sensitive discrimination by *P_DisR* compared to the steady-state displacement used here.

## Conclusion

Discrimination between PSP patients and healthy controls based on vertical saccade velocity or gain can be improved in three ways, namely by (1) using multiplicative combinations of these parameters as discriminators, or (2) using composites of velocity, gain and the displacement rate of SWJ-like patterns of fixational saccades, or (3) checking this displacement rate for extreme values typical of either controls or patients. In the present study, 56% of the controls and 22% of the patients could be identified a priori by the latter method. As our sample sizes were limited (N = 50 each), the improvements reported here are only rough estimates of the improvements that can be expected from more representative samples. This caveat does not invalidate our basic conclusion regarding the benefit of parameter combinations and adjuvant displacement rate checks. However, the discriminative power of parameter combinations in early PSP is lower than in samples that include patients with longer disease duration. Nevertheless, it is hoped that the ability to discriminate between early PSP and Parkinson’s disease can be improved by taking into account the information provided by their fixational saccades. (which will be the target of future studies).

## Data Availability

Upon reasonable request, the data underlying the reported results are available from the corresponding author.

## Abbreviations

AuC: Area under ROC curve
DD: Disease duration
Δ: Magnitude of differences
GM: Geometric mean of specificity and sensitivity
PBF: Paired back-and-forth patterns of SIFS
PSP: Progressive supranuclear palsy
SIFS: Small involuntary fixational saccade
SpcΠ: Point on ROC curve maximising the sensitivity associated with a specificity of Π%
SnsΠ: Point on ROC curve maximising the specificity associated with a sensitivity of Π%
SWJ: Square wave jerks
VGRS: Visually guided reactive saccade
Amp: Global amplitude of SIFS
DisR: Global displacement rate of SIFS
G10: Gain of VGRS evoked by target steps of 10°
*pV*: Maximum achieved peak velocity of VGRS
*P_*: Prefix identifying parameters of PBF patterns
[min max]: Range of values
↔ , ↓, ↑ and ↕: Suffixes indicating direction of VGRS
p1 >< p2: Correlation between parameters p1 and p2

## Footnotes

1 National Institute of Neurological Disorders and Stroke – Society for Progressive Supranuclear Palsy.
